# The different hypoglycemic effects between East Asian and non-Asian type 2 diabetes patients when treated with SGLT-2 inhibitors as an add-on treatment for metformin: a systematic review and meta-analysis of randomized controlled trials

**DOI:** 10.18632/aging.202945

**Published:** 2021-05-11

**Authors:** Xianzhi Li, Qianping Zhang, Xiaojun Zhou, Siyi Guo, Shan Jiang, Yuhan Zhang, Ruzhen Zhang, Jianjun Dong, Lin Liao

**Affiliations:** 1 Department of Endocrinology and Metabology, The First Affiliated Hospital of Shandong First Medical University & Shandong Provincial Qianfoshan Hospital, Shandong Key Laboratory of Rheumatic Disease and Translational Medicine, Shandong Institute of Nephrology, Ji-nan, Shandong 250014, China; 2 Department of Endocrinology and Metabology, Shandong Provincial Qianfoshan Hospital, Shandong University, Shandong Key Laboratory of Rheumatic Disease and Translational Medicine, Shandong Institute of Nephrology, Ji-nan, Shandong 250014, China; 3 Division of Endocrinology, Department of Internal Medicine, Dezhou Municipal Hospital, Dezhou, Shandong 253000, China; 4 Department of Endocrinology, Qilu Hospital, Cheeloo College of Medicine, Shandong University, Ji-nan, Shandong 250012, China

**Keywords:** SGLT-2 inhibitors, metformin, Asian, type 2 diabetes, randomized controlled trial, meta-analysis

## Abstract

Aims: To investigate the efficacy and safety of SGLT-2 inhibitors as an add-on treatment for metformin between Asian and non-Asian T2DM.

Methods: A systematic literature search of PubMed, EMBASE, and the Cochrane Library was performed through August 2020 with the following keywords: Sodium-Glucose Transporter 2 Inhibitors, Sodium Glucose Transporter 2 Inhibitors, SGLT2 inhibitor, SGLT-2 inhibitors, type 2 diabetes, and randomized controlled trials. Double-blinded RCTs comparing SGLT-2 inhibitors as an add-on treatment for metformin and metformin monotherapy in adults with type 2 diabetes were included. A random effects model was used to calculate overall effect sizes.

Results: 5 RCTs with 1193 Asian patients and 7 RCTs with 2098 non-Asian patients were investigated. The improvement in HbA1c and fasting blood glucose in the Asian patients (WMD, −0.73%; 95% CI, −1.01% to −0.46%, *p* < 0.01; WMD, −1.51; 95% CI, −1.81 to −1.21, *p* < 0.01, respectively) were both significantly better than in the non-Asians (WMD, −0.45%; 95% CI, −0.62% to −0.29%, *p* < 0.01; WMD, −1.03; 95% CI, −1.27 to −0.78, *p* < 0.01, respectively). The effect of weight loss was similar in the non-Asian patients and Asian patients. There was little difference in the improvement of systolic blood pressure between them. The risk of serious adverse events was not significantly increased between the Asian and non-Asian patients.

Conclusion: SGLT-2 inhibitors as an add-on treatment for metformin are more efficacious in East Asian T2DM patients than in non-Asian T2DM patients without an additional risk of severe adverse events.

## INTRODUCTION

T2DM is one of the most prevalent diseases in the world and more than half of the patients are in Asia [1, 2]. So, it is important for a hypoglycemic agent to have a good efficacy and safety in Asian T2DM. According to Chinese Diabetes Society guidelines [3], if lifestyle intervention cannot reach the target blood glucose, metformin is often recommended as a first-line pharmacological treatment for patients with T2DM [4, 5]. However, as type 2 diabetes progresses, metformin monotherapy often fails to provide sustained and beneficial glycemic control [6].

The guidelines of both European Association for the Study of Diabetes (EASD) and the American Diabetes Association (ADA) recommend SGLT-2 inhibitors as a combination therapy with other antihyperglycemic drugs at any stage of T2DM and when metformin is not tolerated or ineffective, SGLT-2 inhibitors was recommend as an acceptable alternative to metformin [7–8]. There were similar recommendations in clinical practice guidelines for T2DM in Asian countries [9–11].

SGLT-2 inhibitors are a new type of oral antidiabetic drug that can selectively inhibit renal glucose reabsorption, thereby increasing urinary glucose excretion and reducing hyperglycemia. Furthermore, SGLT-2 inhibitors do not increase the incidence of hypoglycemia because their hypoglycemic mechanism is independent of insulin [12].

At present, as the prevalence of T2DM substantially increases in the Asian population [11], the efficacy and safety of this novel therapeutic regimen for Asian T2DM patients have also attracted increasing attention. There were a series of studies that evaluated the efficacy and safety of the SGLT-2 inhibitors as an add-on treatment for metformin in Asian patients with T2DM [13–17]. However, no studies have been conducted to compare the differences in efficacy and safety of SGLT-2 inhibitors as an add-on treatment for metformin between Asian and non-Asian patients up to now. Due to differences in dietary patterns or habits, body mass index, and genetic and racial backgrounds between Asian and non-Asian population [18], the effect on glycemic control and body weight control as well as the adverse effects of SGLT-2 inhibitors as an add-on treatment for metformin may be different between patients with T2DM in these two populations. Thus, we performed this systematic review and meta-analysis to evaluate the differences in efficacy and safety of this therapeutic regimen between Asian and non-Asian patients with T2DM. Furthermore, we discussed the potential causes of these conflicting results.

## MATERIALS AND METHODS

This systematic review and meta-analysis was conducted and reported according to the Meta-Analysis of Observational Studies in Epidemiology (MOOSE) guidelines [19]. We searched the included studies on the basis of the Preferred Reporting Items for Systematic Reviews and Meta-Analyses (PRISMA) statement [20].

### Databases and search strategy

A systematic literature search was carried out using three online databases, EMBASE, PubMed, and the Cochrane Library, to look for randomized clinical trials directly comparing SGLT-2 inhibitors as an add-on treatment for metformin with metformin monotherapy in patients with T2DM up to August 2020, with no restrictions on the year of publication. The following Medical Subject Headings (MeSH) terms and text words were used in different search combinations: Sodium Glucose Transporter 2 Inhibitors, Sodium-Glucose Transporter 2 Inhibitors, SGLT-2 Inhibitors, SGLT2 inhibitor, type 2 diabetes, and randomized controlled trials.

These articles were qualified by two authors independently reviewing and cross-checking. A manual search was also performed for potentially relevant eligible studies from the reference lists of the included papers. Reviewers resolved their disagreements by consensus.

### Study selection and criteria

Inclusion criteria were as follows: (i) studies on patients with type 2 diabetes; (ii) trials examining SGLT-2 inhibitors as an add-on treatment for metformin by using a metformin monotherapy control group; (iii) trials with a duration greater than 12 weeks; (iv) trials with at least one baseline and posttreatment efficacy or safety outcomes of interest; (v) randomized control trials; and (vi) trials that reported at least one dispersion measure [confidence interval (CI), standard deviation (s.d), or standard error of mean (s.e.m)] for treatment groups of interest.

Exclusion criteria were as follows: (i) studies not published in English; (ii) animal studies; (iii) studies investigating non-SGLT-2 inhibitors as an add-on treatment for metformin interventions; (iv) letters and comments, reviews, and meta-analyses; and (v) studies lacking necessary data for efficacy and safety analyses.

### Data extraction and quality assessment

Two reviewers scanned the studies which qualified for this meta-analysis independently and extracted the corresponding data to a pre-designed form. The reviewers evaluated the study together to reach an agreement, if there was a discrepancy.

The following data were extracted: research characteristics (first author, publication year, trial duration, proportion of Asian subjects, number of study subjects); subjects’ baseline characteristics (average age, sex ratio, diabetes duration, drug dosage, hemoglobin A1c (HbA1c,%), fasting plasma glucose (FPG, mmol/L), body mass index (BMI), systolic blood pressure (SBP, mmHg); outcomes (change in HbA1c, change in FPG, change in body weight (BW, kg), change in SBP, serious adverse events (SAEs, SAEs were any untoward medical events that resulted in death, were life-threatening, resulted in persistent or significant disability/incapacity, required hospitalization or prolonged hospitalization and other medically important events [16]). The same dose groups were selected to achieve maximum equivalence for research that used multiple doses of an SGLT-2 inhibitor [21].

**Figure 1:**
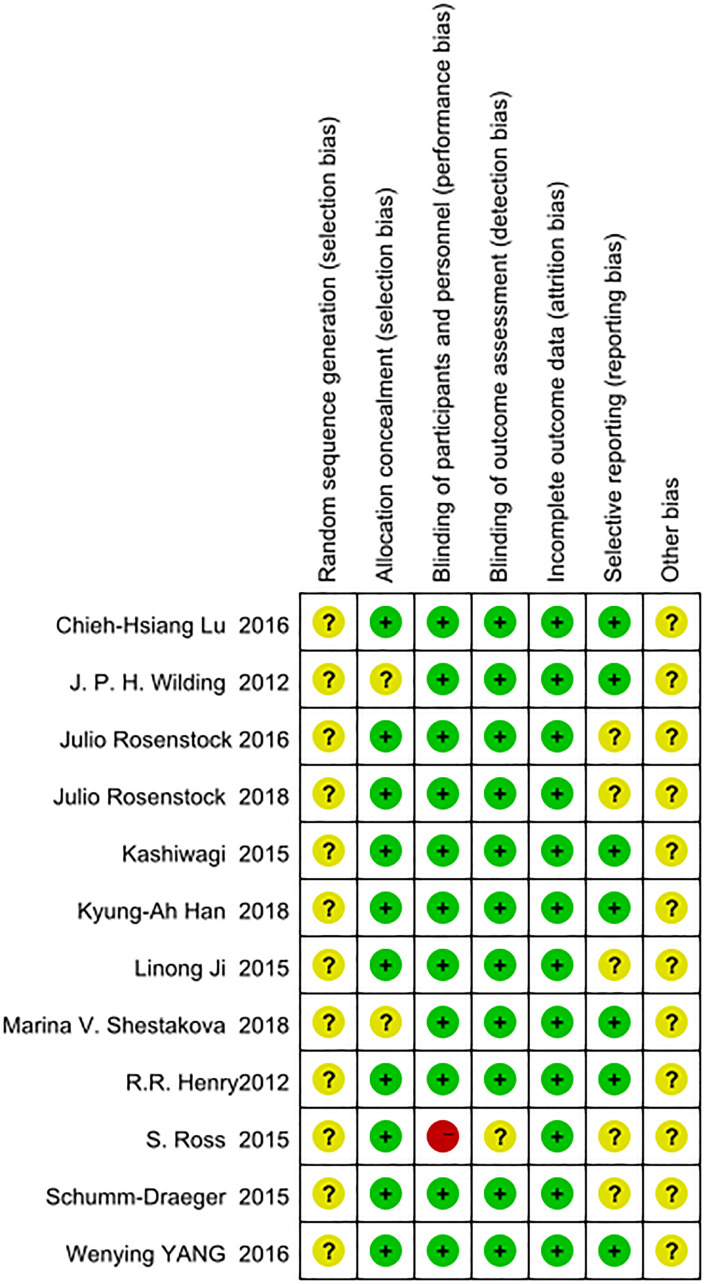
Evaluation of the risk of bias.

We used the Cochrane Collaboration’s risk of bias tool with Review Manager version 5.3 to evaluate the quality of the included RCTs [22]. The quality evaluation criteria included (i) random sequence generation; (ii) allocation concealment; (iii) blinding of subjects and personnel; (iv) blinding of outcome assessment; (v) incomplete outcome data; (vi) selective reporting; and (vii) other bias. Three levels were used to evaluate the risk bias of each study: “high risk”, “unclear risk”, and “low risk”. The results of the evaluations were placed in a risk of bias summary. Details are shown in Figure 1.

### Data synthesis and analysis

The primary outcome was the difference of the two therapeutic regimens in the HbA1c change from baseline between Asian and non-Asian T2DM patients. Other outcomes included (i) change from baseline in FPG; (ii) change from baseline in BW; (iii) change from baseline in SBP; and (iv) incidence of SAEs between Asian and non-Asian T2DM patients.

The meta-analyses with fixed effects were performed by computing odds ratios (ORs) and 95% CIs for the outcomes of dichotomous variables (incidence of SAEs), and weighted mean differences (WMDs) and 95% CIs were used for continuous variables (change from baseline in HbA1c, FPG, BW, and SBP) [23]. Cochran’s Q (chi-squared) test was used to quantify the heterogeneity, and the I^2^ statistic was used to evaluate the extent of inconsistency [24]: I^2^ > 50% was considered substantial heterogeneity [25]. Funnel plots was used to assess publication bias (Supplementary Figures 6, 7). The overall quality of evidence for each outcome was assessed by the Grading of Recommendations Assessment, Development and Evaluation (GRADE) approach (Supplementary Figures 1–5). Analysis of all data was by Review Manager (RevMan 5.3) statistical software and GRADEprofiler software (version 2015, Evidence Prime Inc, McMaster University, Canada).

## RESULTS

### Literature search

**Figure 2:**
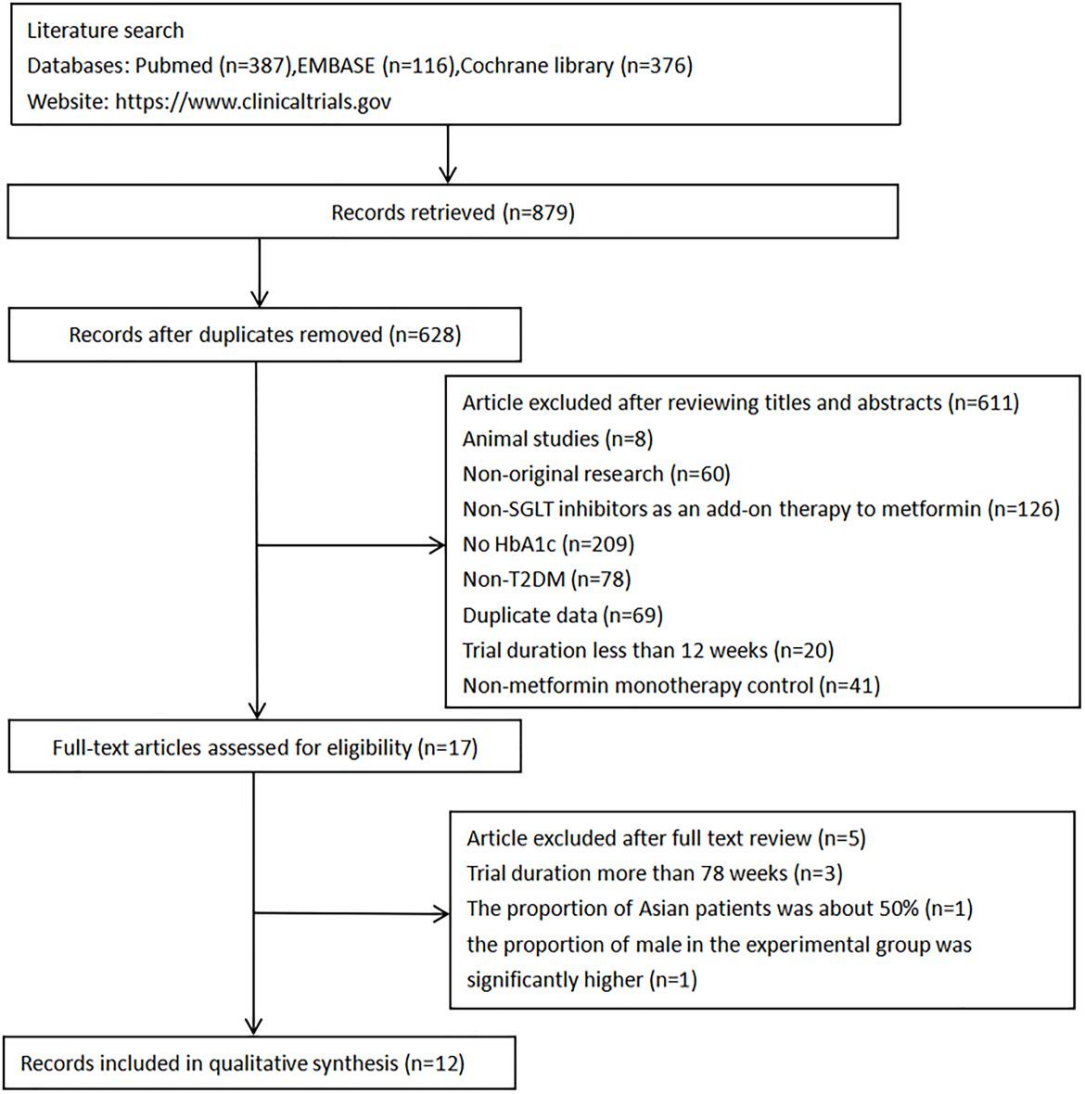
Flow diagram of study selection.

A total of 879 potential articles were identified from the three databases. After removing duplicates, 628 articles remained. Of these, 611records were excluded based on titles and abstracts, resulting in the selection of 17 articles and then a more detailed assessment of their qualifications. Five papers were further discarded for the following reasons: (i) The duration of three studies [26–28] (78–112 weeks) were much longer than the others (12–26 weeks); (ii) The proportion of Asian patients in the paper presented by Merker et al. was about 50%, and it could not be divided into any subgroup [29]; (iii) In the paper presented by Amin et al., the proportion of males in the experimental group was significantly higher than that in the control group, which might affect experimental result [30]. Finally, 12 randomized controlled trials were included. No additional eligible studies were found by searching clinical trial registration website. (the search process is summarized in Figure 2).

### Study characteristics

These 12 selected studies were published from 2010 to 2018 and included a total of 3291 patients with T2DM. The characteristics of the studies are shown in Supplementary Tables 1 and 2. In addition, the Asian subjects in this study mainly refer to patients in East Asia, such as China and Japan.

For Asian subjects, 5 trials (1193 patients) were included [13–17]. Among these, 1 trial involved treatment with canagliflozin, 1 with dapagliflozin, and 3 with ipragliflozin as an add-on treatment for metformin (Supplementary Table 1). All control groups were treated with metformin monotherapy.

For non-Asian subjects, 7 trials (2098 patients) were included [31–37]. Among these, 1 trial used canagliflozin, 2 trials used dapagliflozin, 1 trial used empagliflozin, 2 trials used ertugliflozin and 2 trials used ipragliflozin as an add-on treatment for metformin (Supplementary Table 2). All control groups were treated with metformin monotherapy.

In these trials, the SGLT-2 inhibitors were used at the recommended dose, and metformin was used at no less than 1500 mg daily. The duration of SGLT-2 inhibitors as an add-on treatment for metformin was 12 to 26 weeks.

### Efficacy

SGLT-2 inhibitors significantly improved HbA1c (%) for both Asian and non-Asian subjects as additional treatments for metformin compared with metformin monotherapy. Moreover, the decrease in HbA1c for the Asian patients (WMD, −0.73%; 95% CI, −1.01% to −0.46%, *p* < 0.01) was significantly greater than that for the non-Asian patients (WMD, −0.45%; 95% CI, −0.62% to −0.29%, *p* < 0.01), although with high heterogeneities of I^2^ = 87% and 78%, respectively (Figure 3A). Therefore, a sensitivity analysis by random effect model was conducted to exclude the data leading to significant heterogeneity of the Asian subgroup (Ipragliflozin in combination with metformin for the treatment of Japanese patients with type 2 diabetes [15]) and non-Asian subgroup (Effect of ertugliflozin on glucose control, body weight, blood pressure and bone density in type 2 diabetes mellitus inadequately controlled on metformin monotherapy [35]), and still found that HbA1c was significantly reduced more in Asian patients than in non-Asian patients: (WMD, −0.60; 95% CI, −0.75 to −0.46, *p* < 0.01) vs (WMD, −0.39; 95% CI, −0.51 to −0.27, *p* < 0.01) without a significant heterogeneity (I^2^ = 39% and 49%, respectively). (Figure 3B). Test for subgroup differences: I^2^ = 79.6% (*p* < 0.05). This indicated that the heterogeneity did not affect the results.

**Figure 3A:**
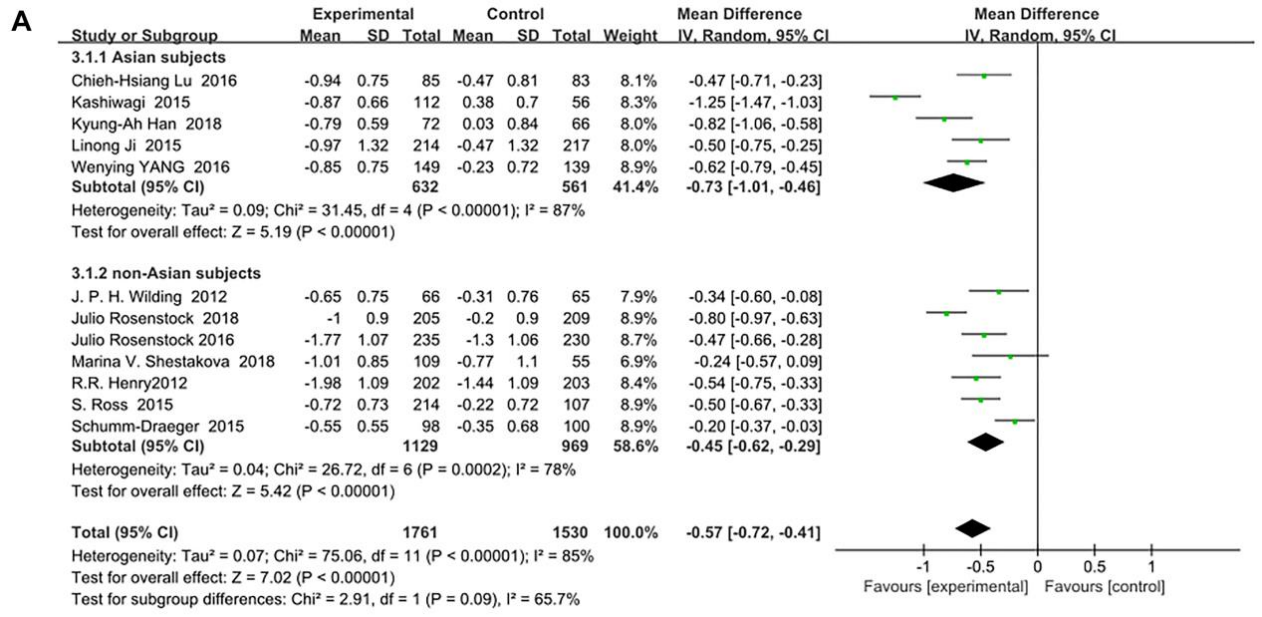
Forest plot of the weighted mean difference in the change of HbA1c from baseline.

**Figure 3B:**
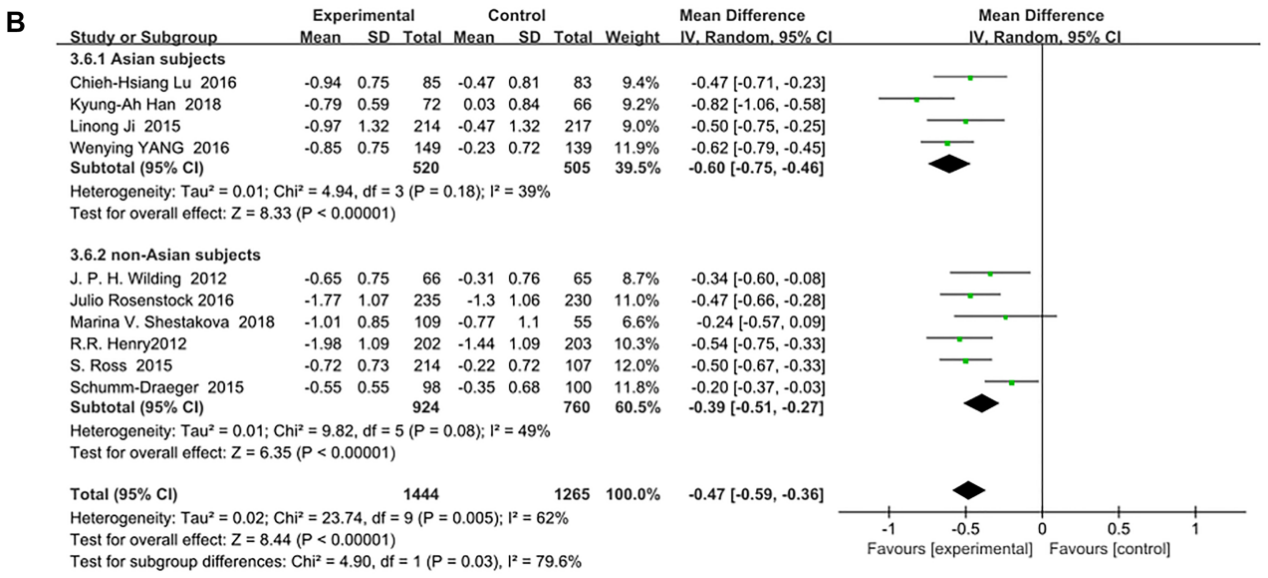
Forest plot of the weighted mean difference in the change of HbA1c from baseline. (sensitivity analysis).

SGLT-2 inhibitors significantly decreased FPG, BW, and SBP when used as additional treatments to metformin compared with metformin monotherapy.

**Figure 4:**
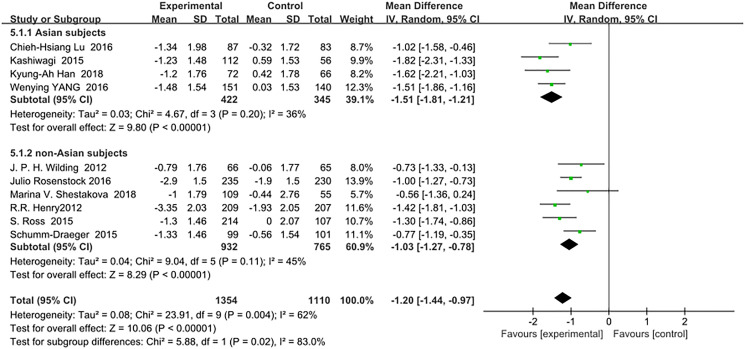
Forest plot of the weighted mean difference in the change of FPG from baseline.

Similarly to the HbA1c results, SGLT-2 inhibitors reduced FPG(mmol/L) significantly more in Asian patients than in non-Asian patients as an add-on treatment for metformin: (WMD, −1.51; 95% CI, −1.81 to −1.21, *p* < 0.01) and (WMD, −1.03; 95% CI, −1.27 to −0.78, *p* < 0.01), respectively (Figure 4). The heterogeneity among all trials in the two subgroups was not significant (I^2^ = 36% and 45%, respectively). Test for subgroup differences: I^2^ = 83% (*p* < 0.01).

**Figure 5:**
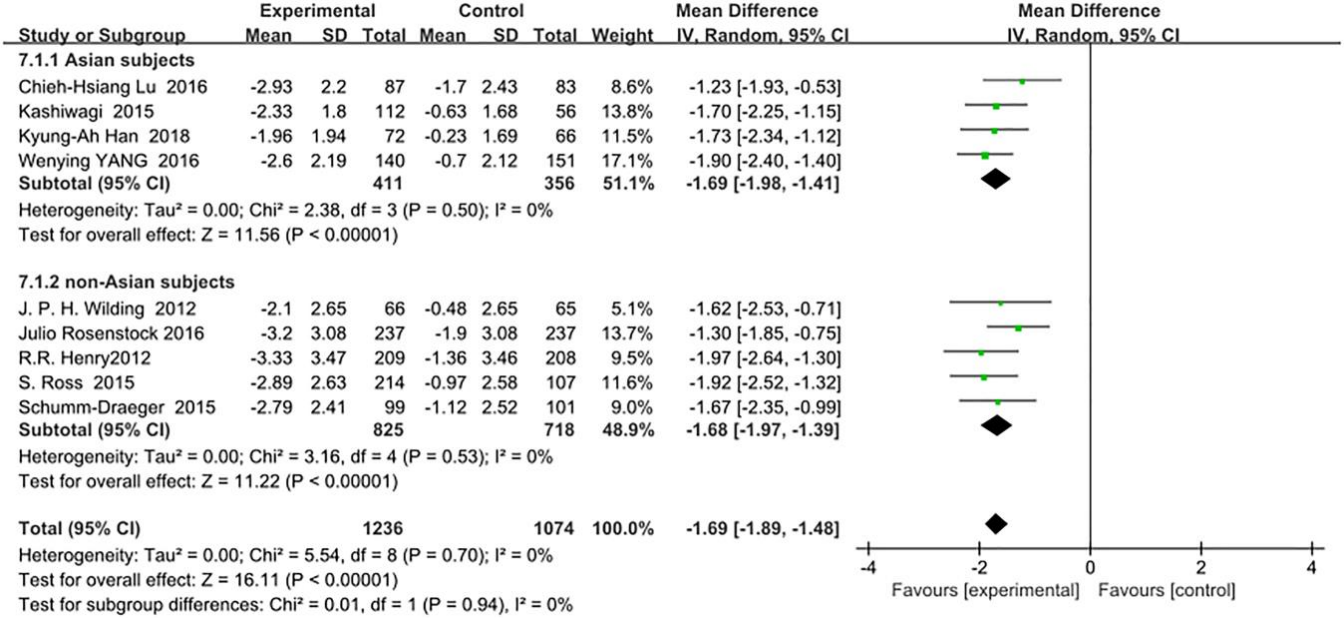
Forest plot of the weighted mean difference in the change of body weight from baseline.

Comparison of body weight changes between Asian and non-Asian T2DM patients corrected by metformin monotherapy indicated no difference between the two groups: (WMD, −1.69; 95% CI, −1.98 to −1.41, *p* < 0.01) vs (WMD, −1.68; 95% CI, −1.97 to −1.39, *p* < 0.01). Details are shown in Figure 5. Test for subgroup differences: I^2^ = 0 (*p* > 0.05).

**Figure 6:**
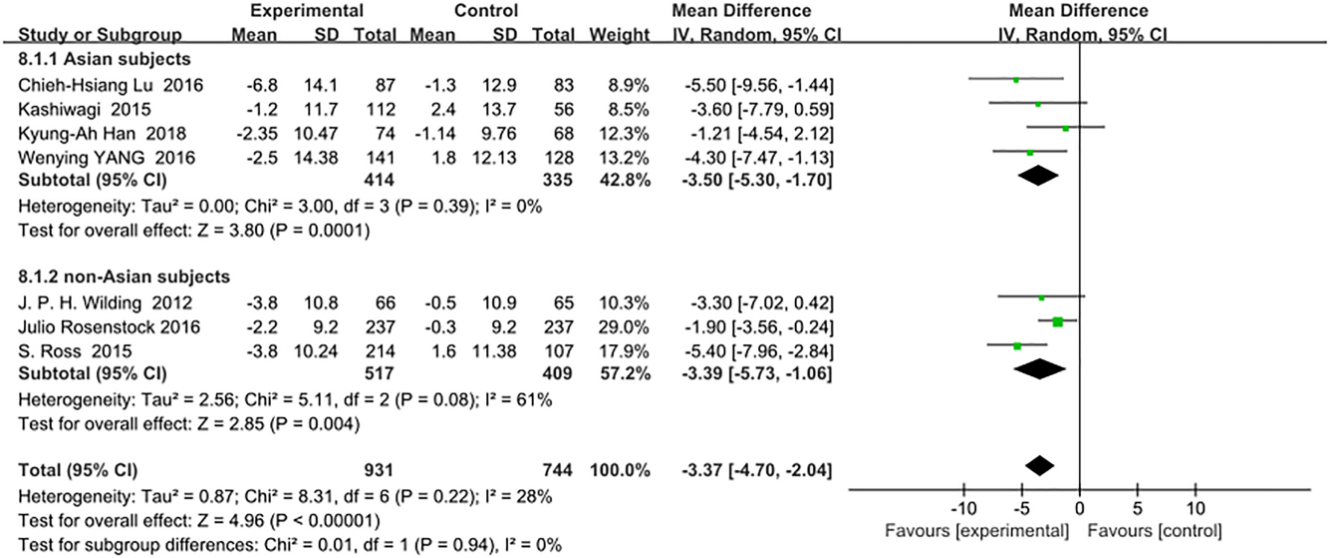
Forest plot of the weighted mean difference in the change of systolic blood pressure from baseline.

There was no significant difference in the reduction of systolic blood pressure (mmHg) between the Asian and non-Asian patients: (WMD, −3.50; 95% CI, −5.30 to −1.70, *p* < 0.01) vs (WMD, −3.39; 95% CI, −5.73 to −1.06, *p* < 0.01), respectively. The total heterogeneity among all trials was not significant (I^2^ = 28%). Test for subgroup differences: I^2^ = 0 (*p* > 0.05). (Figure 6).

### Safety

**Figure 7:**
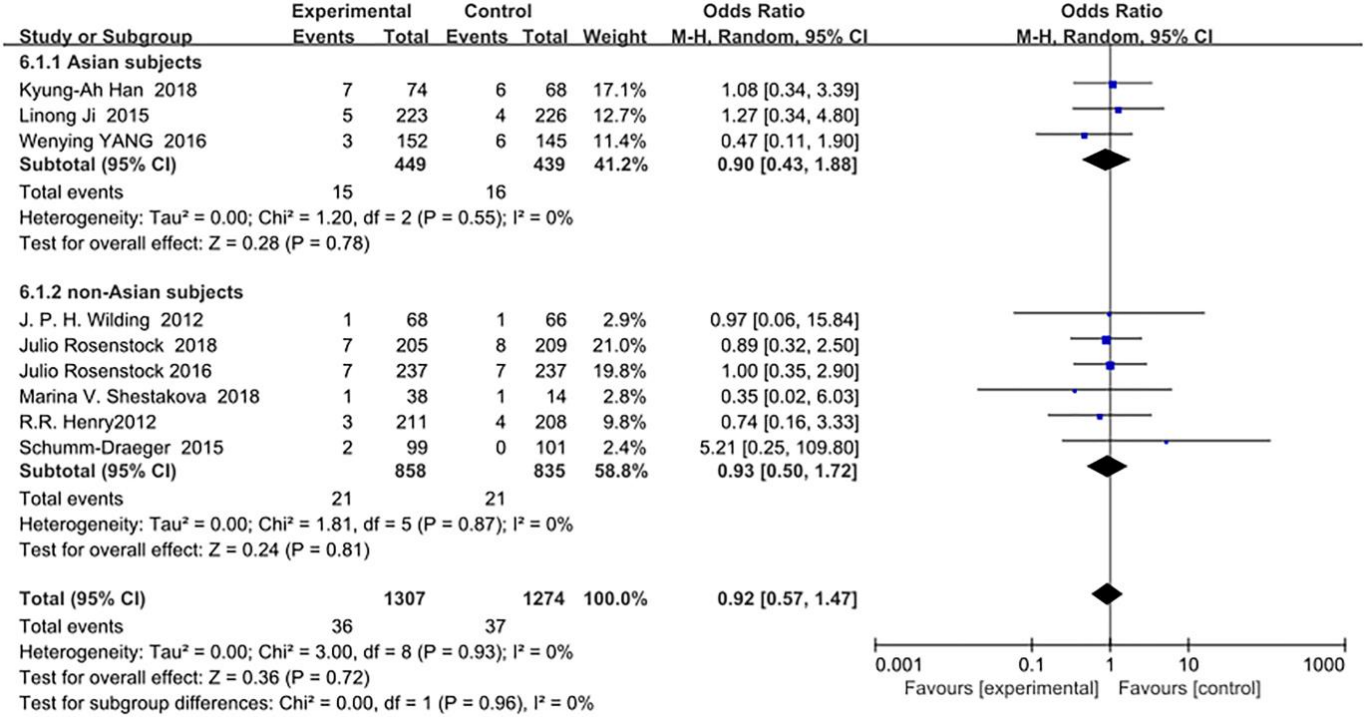
Forest plot of the odds ratio in the incidence of serious adverse events.

SGLT-2 inhibitors as an add-on treatment for metformin did not increase the risk of serious adverse events in Asians (OR = 0.90, 95% CI, 0.43 to 1.88, *p* = 0.78) or in non-Asians (OR = 0.93, 95% CI, 0.50 to 1.72, *p* = 0.81) compared with metformin monotherapy (Figure 7).

## DISCUSSION

Our systematic review evaluated the clinical efficacy and safety of SGLT-2 inhibitors as an add-on treatment for metformin in comparison with metformin monotherapy in Asian and non-Asian T2DM patients. Our results indicated that the improvement in HbA1c and fasting blood glucose in Asian patients were superior to that of non-Asian patients and confirmed the hypothesis that efficacy on glucose control differs between Asian and non-Asian T2DM patients.

The reasons for this discrepancy may lie in the following aspects. First, the body weight of Asians is smaller than non-Asians [38]. So, the drug doses (amount of drug/kg) of SGLT-2 inhibitors are higher in patients of the Asian group. Within a certain range, the hypoglycemic effects of SGLT-2 inhibitors are positively correlated with the dose [39, 40]. The second reason might be the different genetic and ethnic backgrounds of Asians and non-Asians, Asians have higher insulin resistance than non-Asians [41, 42] and SGLT2 inhibitors could attenuates inflammation and insulin resistance [43, 44], and could improve insulin resistance more in Asians than non-Asians. Besides, several biological differences have been identified between patients of Asian and non-Asian patients, which might cause the difference in pharmacokinetics and pharmacodynamics of SGLT-2 inhibitor between the two groups [45].

The weight decrease corrected by metformin monotherapy for the non-Asian patients was similar to that for the Asian patients. The mechanism of weight loss caused by SGLT-2 inhibitors may be due to glycosuria, which in turn leads to energy as well as water loss through osmotic diuresis [46, 47]. Another possible cause is the reduction in total body adipose tissue volume, which similarly involves subcutaneous and visceral deposits [48].

In addition, both in Asian and non-Asian T2DM patients, SBP decreased significantly following treatment with SGLT-2 inhibitors as an add-on treatment for metformin, and there was no significant difference between the two groups. According to current data, the decrease in blood pressure may be due to the combination of diuresis, nephron remodeling, and weight loss [47, 49].

Regarding safety, the incidence of serious adverse events from SGLT-2 inhibitors as an add-on treatment for metformin, both in Asian and non-Asian T2DM patients, indicated no significant change compared with metformin monotherapy. There was also no significant difference in the incidence of severe adverse events between the Asian and non-Asian T2DM patients with SGLT-2 inhibitors as an add-on treatment for metformin [50, 51].

There are limitations in this systematic review and meta-analysis. First, the trials investigating non-Asian individuals recruited Asians may cause bias in results, and we discarded trials whose proportion of Asian patients more than 20% (eg., Merker et al. was about 50% [29]) from non-Asian group to minimize this bias. Second, only Chinese and Japanese subjects (5 studies with 1193 subjects) were included since trial from other Asian countries was not found from the databases, which may be diverse from China and Japan population and may influence the outcome of the study, we therefore labeled “Asian patients” as “East Asian patients” in the conclusion to minimize the possible bias. Third, the duration of SGLT-2 inhibitors as an add-on treatment for metformin in this meta-analysis was 12 to 26 weeks, which was short-term and the long-term effect needs further study. Fourth, the statistical heterogeneity (I²) was relatively high for the comparison of the change in HbA1c (I² = 87% and 78%, respectively). The different types of SGLT-2 inhibitors used and the different treatment times may have contributed to the high level of heterogeneity. Because of the high heterogeneity, we used random effect model to conduct a sensitivity analysis excluding the data leading to significant heterogeneity of each group. Moreover, the findings may be restricted to some brands of SGLT-2 inhibitors, because SGLT-2 inhibitors used in East Asian patients were canagliflozin, dapagliflozin and ipragliflozin, while in non-Asian patients were canagliflozin, dapagliflozin, ipragliflozin, empagliflozin and ertugliflozin. Therefore, given the above limitations, our results should be interpreted with caution.

## CONCLUSIONS

According to this systematic review and meta-analysis, when SGLT-2 inhibitors are used as an add-on treatment for metformin, the decrease in HbA1c and fasting blood glucose in East Asian T2DM patients was significantly greater than in non-Asian patients compared with metformin monotherapy. There was no significant difference in the change in systolic blood pressure and body weight between the two groups.

Moreover, there was no significant difference in severe adverse events between the East Asian and non-Asian T2DM patients when treated with SGLT-2 inhibitors as an add-on treatment for metformin. Therefore, this treatment regimen could be more efficacious in East Asian T2DM patients than in non-Asian T2DM patients in short term without an additional risk of severe adverse events. Additional clinical trials with larger sample sizes and more available data with different races may better clarify these issues.

## Supplementary Materials

Supplementary Figures

Supplementary Table 1

Supplementary Table 2
