# Supplementary material for: The different hypoglycemic effects between East Asian and non-Asian type 2 diabetes patients when treated with SGLT-2 inhibitors as an add-on treatment for metformin: a systematic review and meta-analysis of randomized controlled trials
Source: Aging (Albany NY). 2021 May 11;13(9):12748–65. doi: 10.18632/aging.202945 (PMC8148508; doi:10.18632/aging.202945)
Supplement: Supplementary Figures [file aging-13-202945-s001.pdf]

SUPPLEMENTARY FIGURES

SGLT2-i plus MET compared to MET monotherapy for T2DM

Patient or population: patients with T2DM
Settings:
Intervention: SGLT2-i plus MET
Comparison: MET monotherapy

Outcomes	Illustrative comparative risks* (95% CI) Assumed risk MET monotherapy	Corresponding risk SGLT2-i plus MET	Relative effect (95% CI)	No of Participants (studies)	Quality of the evidence (GRADE)	Comments
**HbA1c (sensitivity analysis)** HbA1c Follow-up: 12-26 weeks		The mean hba1c (sensitivity analysis) in the intervention groups was **0.47 lower** (0.54 to 0.41 lower)		2709 (10 studies)	⊕⊕⊕⊖ **moderate**[1]	
**HbA1c (sensitivity analysis) - Asian subjects** HbA1c Follow-up: 18-24 weeks		The mean hba1c (sensitivity analysis) - asian subjects in the intervention groups was **0.61 lower** (0.71 to 0.5 lower)		1025 (4 studies)	⊕⊕⊕⊖ **moderate**[1]	
**HbA1c (sensitivity analysis) - non-Asian subjects** HbA1c Follow-up: 12-26 weeks		The mean hba1c (sensitivity analysis) - non-asian subjects in the intervention groups was **0.4 lower** (0.48 to 0.31 lower)		1684 (6 studies)	⊕⊕⊕⊖ **moderate**[1]	

*The basis for the **assumed risk** (e.g. the median control group risk across studies) is provided in footnotes. The **corresponding risk** (and its 95% confidence interval) is based on the assumed risk in the comparison group and the **relative effect** of the intervention (and its 95% CI).

CI: Confidence interval;

GRADE Working Group grades of evidence
High quality: Further research is very unlikely to change our confidence in the estimate of effect.
Moderate quality: Further research is likely to have an important impact on our confidence in the estimate of effect and may change the estimate.
Low quality: Further research is very likely to have an important impact on our confidence in the estimate of effect and is likely to change the estimate.
Very low quality: We are very uncertain about the estimate.

[1] Funnel diagrams of HbA1c

Supplementary Figure 1. GRADE approach to assess the overall confidence for HbA1c.

SGLT2-i plus MET compared to MET monotherapy for T2DM

Patient or population: patients with T2DM
Settings:
Intervention: SGLT2-i plus MET
Comparison: MET monotherapy

Outcomes	Illustrative comparative risks* (95% CI) Assumed risk MET monotherapy	Corresponding risk SGLT2-i plus MET	Relative effect (95% CI)	No of Participants (studies)	Quality of the evidence (GRADE)	Comments
**FPG** FPG Follow-up: 12-26 weeks		The mean fpg in the intervention groups was **1.2 lower** (1.34 to 1.07 lower)		2464 (10 studies)	⊕⊕⊕⊕ **high**	
**FPG - Asian subjects** FPG Follow-up: 18-24 weeks		The mean fpg - asian subjects in the intervention groups was **1.51 lower** (1.75 to 1.28 lower)		767 (4 studies)	⊕⊕⊕⊕ **high**	
**FPG - non-Asian subjects** FPG Follow-up: 12-26 weeks		The mean fpg - non-asian subjects in the intervention groups was **1.04 lower** (1.21 to 0.88 lower)		1697 (6 studies)	⊕⊕⊕⊕ **high**	

*The basis for the **assumed risk** (e.g. the median control group risk across studies) is provided in footnotes. The **corresponding risk** (and its 95% confidence interval) is based on the assumed risk in the comparison group and the **relative effect** of the intervention (and its 95% CI).

CI: Confidence interval;

GRADE Working Group grades of evidence
High quality: Further research is very unlikely to change our confidence in the estimate of effect.
Moderate quality: Further research is likely to have an important impact on our confidence in the estimate of effect and may change the estimate.
Low quality: Further research is very likely to have an important impact on our confidence in the estimate of effect and is likely to change the estimate.
Very low quality: We are very uncertain about the estimate.

Supplementary Figure 2. GRADE approach to assess the overall confidence for FPG.

SGLT2-i plus MET compared to MET monotherapy for T2DM

Patient or population: patients with T2DM
Settings:
Intervention: SGLT2-i plus MET
Comparison: MET monotherapy

Outcomes	Illustrative comparative risks* (95% CI)		Relative effect (95% CI)	No of Participants (studies)	Quality of the evidence (GRADE)	Comments
	Assumed risk	Corresponding risk				
	MET monotherapy	SGLT2-i plus MET				
**Body Weight**<br>Body Weight<br>Follow-up: 12-26 weeks		The mean body weight in the intervention groups was<br>**1.69 lower**<br>(1.89 to 1.48 lower)		2310<br>(9 studies)	⊕⊕⊕⊕<br>**high**	
**Body Weight - Asian subjects**<br>Body Weight<br>Follow-up: 18-24 weeks		The mean body weight - asian subjects in the intervention groups was<br>**1.69 lower**<br>(1.98 to 1.41 lower)		767<br>(4 studies)	⊕⊕⊕⊕<br>**high**	
**Body Weight - non-Asian subjects**<br>Body Weight<br>Follow-up: 12-26 weeks		The mean body weight - non-asian subjects in the intervention groups was<br>**1.68 lower**<br>(1.97 to 1.39 lower)		1543<br>(5 studies)	⊕⊕⊕⊕<br>**high**	

*The basis for the **assumed risk** (e.g. the median control group risk across studies) is provided in footnotes. The **corresponding risk** (and its 95% confidence interval) is based on the assumed risk in the comparison group and the **relative effect** of the intervention (and its 95% CI).

CI: Confidence interval;

GRADE Working Group grades of evidence
High quality: Further research is very unlikely to change our confidence in the estimate of effect.
Moderate quality: Further research is likely to have an important impact on our confidence in the estimate of effect and may change the estimate.
Low quality: Further research is very likely to have an important impact on our confidence in the estimate of effect and is likely to change the estimate.
Very low quality: We are very uncertain about the estimate.

Supplementary Figure 3. GRADE approach to assess the overall confidence for Body Weight.

SGLT2-i plus MET compared to MET monotherapy for T2DM

Patient or population: patients with T2DM
Settings:
Intervention: SGLT2-i plus MET
Comparison: MET monotherapy

Outcomes	Illustrative comparative risks* (95% CI)		Relative effect (95% CI)	No of Participants (studies)	Quality of the evidence (GRADE)	Comments
	Assumed risk	Corresponding risk				
	MET monotherapy	SGLT2-i plus MET				
**SBP**<br>SBP<br>Follow-up: 12-26 weeks		The mean sbp in the intervention groups was<br>**3.16 lower**<br>(4.21 to 2.1 lower)		1675<br>(7 studies)	⊕⊕⊕⊕<br>**high**	
**SBP - Asian subjects**<br>SBP<br>Follow-up: 18-24 weeks		The mean sbp - asian subjects in the intervention groups was<br>**3.5 lower**<br>(5.3 to 1.7 lower)		749<br>(4 studies)	⊕⊕⊕⊕<br>**high**	
**SBP - non-Asian subjects**<br>SBP<br>Follow-up: 12-26 weeks		The mean sbp - non-asian subjects in the intervention groups was<br>**2.98 lower**<br>(4.28 to 1.68 lower)		926<br>(3 studies)	⊕⊕⊕⊕<br>**high**	

*The basis for the **assumed risk** (e.g. the median control group risk across studies) is provided in footnotes. The **corresponding risk** (and its 95% confidence interval) is based on the assumed risk in the comparison group and the **relative effect** of the intervention (and its 95% CI).

CI: Confidence interval;

GRADE Working Group grades of evidence
High quality: Further research is very unlikely to change our confidence in the estimate of effect.
Moderate quality: Further research is likely to have an important impact on our confidence in the estimate of effect and may change the estimate.
Low quality: Further research is very likely to have an important impact on our confidence in the estimate of effect and is likely to change the estimate.
Very low quality: We are very uncertain about the estimate.

Supplementary Figure 4. GRADE approach to assess the overall confidence for SBP.

SGLT2-i plus MET compared to MET monotherapy for T2DM

Patient or population: patients with T2DM
Settings:
Intervention: SGLT2-i plus MET
Comparison: MET monotherapy

Outcomes	Illustrative comparative risks* (95% CI) Assumed risk MET monotherapy	Corresponding risk SGLT2-i plus MET	Relative effect (95% CI)	No of Participants (studies)	Quality of the evidence (GRADE)	Comments
**Serious Adverse events** SAEs Follow-up: 12-26 weeks	**Study population**		**OR 0.93** (0.59 to 1.48)	2581 (9 studies)	⊕⊕⊕⊕ **high**	
	**29 per 1000**	**27 per 1000** (17 to 42)				
	**Moderate**					
	**30 per 1000**	**28 per 1000** (18 to 44)				
**Serious Adverse events - Asian subjects** SAEs Follow-up: 18-24 weeks	**Study population**		**OR 0.89** (0.43 to 1.84)	888 (3 studies)	⊕⊕⊕⊕ **high**	
	**36 per 1000**	**33 per 1000** (16 to 65)				
	**Moderate**					
	**41 per 1000**	**37 per 1000** (18 to 73)				
**Serious Adverse events - non-Asian subjects** SAEs Follow-up: 12-26 weeks	**Study population**		**OR 0.96** (0.52 to 1.76)	1693 (6 studies)	⊕⊕⊕⊕ **high**	
	**25 per 1000**	**24 per 1000** (13 to 43)				
	**Moderate**					
	**24 per 1000**	**23 per 1000** (13 to 41)				

*The basis for the **assumed risk** (e.g. the median control group risk across studies) is provided in footnotes. The **corresponding risk** (and its 95% confidence interval) is based on the assumed risk in the comparison group and the **relative effect** of the intervention (and its 95% CI).

CI: Confidence interval; **OR:** Odds ratio;

GRADE Working Group grades of evidence
High quality: Further research is very unlikely to change our confidence in the estimate of effect.
Moderate quality: Further research is likely to have an important impact on our confidence in the estimate of effect and may change the estimate.
Low quality: Further research is very likely to have an important impact on our confidence in the estimate of effect and is likely to change the estimate.
Very low quality: We are very uncertain about the estimate.

Supplementary Figure 5. GRADE approach to assess the overall confidence for SAEs.

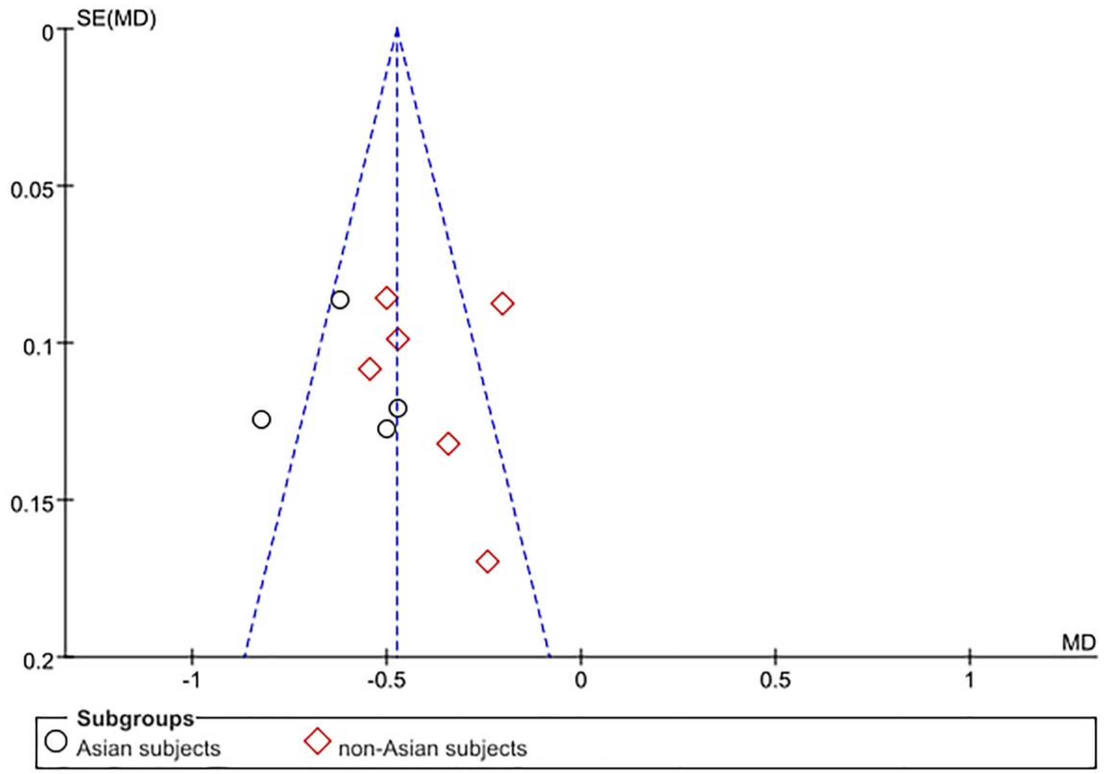

Supplementary Figure 6. Funnel diagrams of HbA1c.

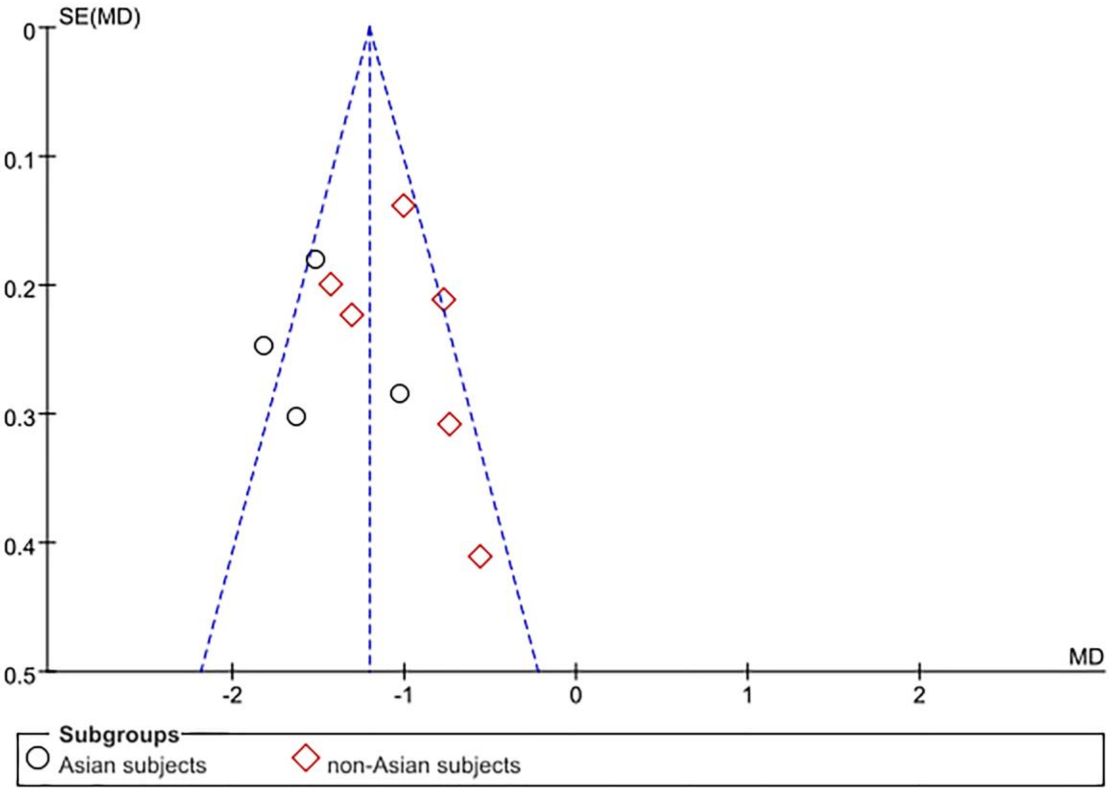

Supplementary Figure 7. Funnel diagrams of FPG.
