# Supplementary material for: The different hypoglycemic effects between East Asian and non-Asian type 2 diabetes patients when treated with SGLT-2 inhibitors as an add-on treatment for metformin: a systematic review and meta-analysis of randomized controlled trials
Source: Aging (Albany NY). 2021 May 11;13(9):12748–65. doi: 10.18632/aging.202945 (PMC8148508; doi:10.18632/aging.202945)
Supplement: Supplementary Table 1 [file aging-13-202945-s002.doc]

**Supplementary Table 1. Characteristics of the randomized controlled trials included in the Asian subgroup.**

| First author Trial duration Asian Diabetes duration Age Male (%) Number of Dosage HbA1c at baseline (%) BMI at baseline，kg/㎡ FPG, mmol/L  (year) (weeks) patients (years) (years) patients  SGLT2-i MET SGLT2-i MET SGLT2-i MET SGLT2-i MET SGLT2-i MET SGLT2-i MET SGLT2-i MET SGLT2-i MET SGLT2-i MET  plus MET monotherapy plus MET monotherapy plus MET monotherapy plus MET monotherapy plus MET monotherapy (mg/day) (mg/day) plus MET monotherapy plus MET monotherapy plus MET monotherapy |
| --- |
| Linong Ji 2015 18 100% 100% 6.8±4.5 6.8±4.5 56.5±8.3 55.8±9.4 55.6 55.3 223 226 CANA 100 ≥1500 8.0±0.9 7.9±0.9 25.6±3.4 25.5±3.6 8.7±1.9 8.8±1.8 |
| Wenying YANG 2016 24 100% 100% 5.3±4.6 5.3±4.4 54.6±9.5 53.5±9.2 57.9 59.3 152 145 DAPA 10 ≥1500 8.17±0.84 8.13±0.85 26.2±3.5 25.7 ± 2.9 9.0 ± 2.2 9.2±2.5 |
| Kashiwagi 2015 24 100% 100% 7.49±5.67 8.05±5.16 56.2±10.7 57.7±9.24 58.9 58.9 112 56 IPRA 50 ≥1500 8.25±0.72 8.38±0.74 25.96±4.4 25.5±3.09 8.98±1.66 9.70±1.38 |
| Chieh-Hsiang Lu 2016 24 100% 100% --------- -------- -------- -------- ------- ------- ------- ------- IPRA 50 ≥1500 -------- -------- -------- -------- -------- -------- |
| Kyung-Ah Han 2018 24 100% 100% 11.6±5.9 11.33±6.6 57.6±8.26 57.4±7.88 50.7 48.5 73 66 IPRA 50 ≥1500 7.90±0.69 7.92±0.79 25.5±3.07 26.1±3.8 8.77±1.6 8.85±1.84 |

CANA, canagliflozin; DAPA, dapagliflozin; EMPA, empagliflozin; ERTU, ertugliflozin; IPRA, ipragliflozin; MET, metformin;

BMI, body mass index; FPG, fasting plasma glucose;Data are mean ± SD or mean; SD, standard deviation.
