# Supplementary material for: The different hypoglycemic effects between East Asian and non-Asian type 2 diabetes patients when treated with SGLT-2 inhibitors as an add-on treatment for metformin: a systematic review and meta-analysis of randomized controlled trials
Source: Aging (Albany NY). 2021 May 11;13(9):12748–65. doi: 10.18632/aging.202945 (PMC8148508; doi:10.18632/aging.202945)
Supplement: Supplementary Table 2 [file aging-13-202945-s003.doc]

**Supplementary Table 2. Characteristics of the randomized controlled trials included in the non-Asian subgroup.**

| First author Trial duration Diabetes duration Age Male (%) Number of Dosage HbA1c at baseline (%) BMI at baseline，kg/㎡ FPG, mmol/L  (year) (weeks) (years) (years) patients  SGLT2-i MET SGLT2-i MET SGLT2-i MET SGLT2-i MET SGLT2-i MET SGLT2-i MET SGLT2-i MET SGLT2-i MET  plus MET monotherapy plus MET monotherapy plus MET monotherapy plus MET monotherapy (mg/day) (mg/day) plus MET monotherapy plus MET monotherapy plus MET monotherapy |
| --- |
| Julio Rosenstock 2016  26 2.9±3.3 3.3±4.5 54.2 ±9.6 55.2 ±9.8   45.6 48.9   235 230 CANA 100 ≥1500 8.8±1.1 8.8±1.2 31.9±5.3 33.0±6.0 10.6±2.8 10.6±2.7 |
| S. Ross 2015 16 -------- ------- -------- -------- ----- ----- 214 107 EMPA 25 ≥1500 7.73±0.05 7.69±0.07 -------- -------- 8.7±0.1 8.9±0.2 |
| N. B. Amin 2015 12 6.0 6.4 54.2±8.8 54±8.1 67.3 55.6 55 54 ERTU 25 <3000 8.30±0.16 8.08±0.14 29.8±0.67 30.6±0.61 9.52±0.43 9.18±0.31 |
| Julio Rosenstock 2018 26 8.1±5.5 8.0±6.3 56.9±9.4 56.5±8.7 45.4 46.9 205 209 ERTU 15 ≥1500 8.1±0.9 8.2±0.9 31.1±4.5 30.7±4.7 9.3±2.5 9.4±2.3 |
| Marina V. 2018 12 6.1±5.9 5.8±5.3 58.9±9.3 58.0±9.5 43.6 40.0 110 55 IPRA 50 ≥1500 8.39±0.93 8.46±0.96 32.8±4.76 31.95±4.2 9.49±2.25 9.76±2.4 |
| J.P. H. Wilding 2012 12 6.0±5.3 5.7±3.2 58.6±7.6 57.3±8.6 47.1 54.5 68 66 IPRA 50 ≥1500 7.76±0.66 7.68±0.60 31.1±4.9 32.0±4.8 8.5±2.0 8.6±1.5 |
| R.R.Henry 2012 24 2.2±3.3 1.9±4.0 51.0±10.1 51.0±10.1  50.2 46.6 211 208 DAPA 10 ≥1500 9.1±1.3 9.1±1.3   --------- --------- 10.52±3.22 10.57±3.0 |
| Schumm-Draeger 2015 16 5.12±4.2 5.53±4.23 55.3±9.3 58.5±9.4 46.5 46.5 99 101 DAPA 10 ≥1500 7.78±0.76 7.94±0.85 33.09±4.94 31.74±4.7 8.62±1.77 8.76±1.99 |

CANA, canagliflozin; DAPA, dapagliflozin; EMPA, empagliflozin; ERTU, ertugliflozin; IPRA, ipragliflozin; MET, metformin;

BMI, body mass index; FPG, fasting plasma glucose;Data are mean ± SD or mean; SD, standard deviation.
